# Differential research impact in cancer practice guidelines’ evidence base: lessons from ESMO, NICE and SIGN

**DOI:** 10.1136/esmoopen-2017-000258

**Published:** 2018-01-06

**Authors:** Elena Pallari, Anthony W Fox, Grant Lewison

**Affiliations:** 1 Health Service and Population Research Department, Centre for Implementation Science, Institute of Psychiatry, Psychology & Neuroscience, King’s College London, London, UK; 2 Institute of Pharmaceutical Sciences, Academic Centre, King’s College London, London, UK; 3 Division of Cancer Studies, Research Oncology, Institute of Cancer Policy, Guy’s Hospital, King’s College London, London, UK

**Keywords:** cancer, clinical practice guidelines, research impact, evidence-base, Esmo, Nice, Sign

## Abstract

**Background:**

This is an appraisal of the impact of cited research evidence underpinning the development of cancer clinical practice guidelines (CPGs) by the professional bodies of the European Society for Medical Oncology (ESMO), the National Institute for Health and Care Excellence (NICE) and the Scottish Intercollegiate Guidelines Network (SIGN).

**Methods:**

A total of 101 CPGs were identified from ESMO, NICE and SIGN websites across 13 cancer sites. Their 9486 cited references were downloaded from the Web of Science Clarivate Group database, analysed on Excel (2016) using Visual Basic Application macros and imported onto SPSS (V.24.0) for statistical tests.

**Results:**

ESMO CPGs mostly cited research from Western Europe, while the NICE and SIGN ones from the UK, Canada, Australia and Scandinavian countries. The ESMO CPGs cited more recent and basic research (eg, drugs treatment), in comparison with NICE and SIGN CPGs where older and more clinical research (eg, surgery) papers were referenced. This chronological difference in the evidence base is also in line with that ESMO has a shorter gap between the publication of the research and its citation on the CPGs. It was demonstrated that ESMO CPGs report more chemotherapy research, while the NICE and SIGN CPGs report more surgery, with the results being statistically significant.

**Conclusions:**

We showed that ESMO, NICE and SIGN differ in their evidence base of CPGs. Healthcare professionals should be aware of this heterogeneity in effective decision-making of tailored treatments to patients, irrespective of geographic location across Europe.

**Key questions**

**What is already known about this subject?**

- Use of clinical practice guidelines (CPGs) has been shown to change clinical practice and improve the quality of patient care.
- Recommendations on CPGs should be based on the best available evidence; however, heterogeneity between development bodies has been demonstrated before.
- In this work, the research evidence base underpinning all the oncology CPGs published by European Society for Medical Oncology (ESMO), National Institute for Health and Care Excellence (NICE) and Scottish Intercollegiate Guidelines Network (SIGN) was assessed.

**What does this study add?**

- The differences are on national origins, time elapsed from publication to citation, research level (ie, basic or clinical) and research domain (eg, surgery, chemotherapy).
- The USA was the biggest contributor to the citations in the evidence base across all oncology CPGs.
- The ESMO CPGs cite mostly more basic research from Western Europe focusing on new chemotherapy agents or targeted treatments.
- The UK NICE and SIGN cite more clinical research from the UK, Canada, Australia and Scandinavian countries reporting more surgery.

**How might this impact on clinical practice?**

- This chronological difference in the evidence base is also in line with that ESMO has a shorter gap between the publication of the research and its citation on the CPGs.
- A closer collaboration between these professional bodies can lead to the use of more evidence-based, relevant and updated CPGs.
- Healthcare professionals using CPGs to personalise treatment regimens should be aware of these biases.

## Introduction

Clinical practice guidelines (CPGs) in oncology inform this expensive medical practice for the clinical care of patients with cancer. Use of CPGs has been shown to change clinical practice and improve the quality of patient care.^1–3^

The European Society for Medical Oncology (ESMO) was founded in 1975 and comprises a range of oncology stakeholders from 130 countries.^4^ In the UK, the National Institute for Health and Care Excellence (NICE), set up in 1999, provides evidence-based recommendations on healthcare practice in England and Wales.^5^ The Scottish Intercollegiate Guidelines Network (SIGN) was set up in 1993 by the Academy of Royal Colleges for the National Health Service in Scotland^6^ with wider consultation from professional and patient representative groups.^7^

Recommendations on CPGs should be based on the best available evidence.^8 9^ An assessment of the research evidence base underpinning recommendations is important, as it demonstrates the degree of medical research transfer into practice.^10–13^ The mere assessment of the contribution of research papers by counting citations in the peer-reviewed literature^10^ does not reflect the impact of assimilation into clinical guidelines or implementation in care.^11 14 15^ The use of bibliometric analysis of CPGs addresses these gaps in understanding research activities, funding agenda and international collaboration in the cancer research arena, and guides public health policy.^16–18^

Pentheroudakis *et al*^19^ challenged the research and clinical community to rethink CPGs’ heterogeneity almost a decade ago. Are CPG developers using similar evidence bases? Or are these different interpretations of similar evidence bases? Here, we evaluate the evidence base of oncology CPGs and consider the clinical impact they could have on the CPGs developed by ESMO, NICE and SIGN.

## Methods

**Figure 1:**
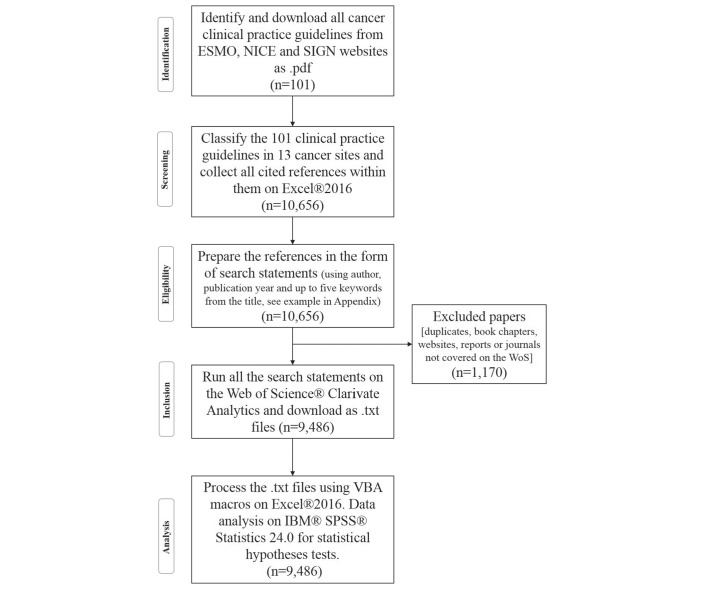
Flow chart of the used methodology adapted from Moher *et al.*^38^ ESMO, European Society for Medical Oncology; NICE, National Institute for Health and Care Excellence; SIGN, Scottish Intercollegiate Guidelines Network.

Oncology CPGs (n*=*101) were collected from the ESMO,^20^ NICE^21^ and SIGN^22^ websites and downloaded, with a cut-off date of 8 April 2017 (for the methodology, see figure 1). For their citation details, see online supplementary appendix A – bibliography. Oncology CPGs were then classified using the ESMO system; this system has 13 major cancer types based on anatomical region or organ system (see table 1).

10.1136/esmoopen-2017-000258.supp1 Supplementary file 1

**Table 1:** The list of the cancer sites is presented in alphabetical order, as classified according to topic and title of each of the identified cancer CPGs (based on the cancer anatomy classification)

No.	Cancer sites (CPG topic)	ESMO	NICE and SIGN	All
1	Breast cancer	4 (n*=*271)	4 (n*=*966)	8 (n*=*1237)
2	Cancers of unknown primary site	1 (n*=*25)	2 (n*=*217)	3 (n*=*242)
3	CNS malignancies	1 (n*=*68)	N/A (n*=*0)	1 (n*=*68)
4	Endocrine and neuroendocrine cancers	4 (n*=*113)	N/A (n*=*0)	4 (n*=*113)
5	Gastrointestinal cancers	11 (n*=*522)	5 (n*=*788)	14 (n*=*1310)
6	Genitourinary cancers	6 (n*=*385)	4 (n*=*1334)	10 (n*=*1719)
7	Gynaecological cancers	5 (n*=*185)	3 (n*=*486)	8 (n*=*671)
8	Haematological malignancies	14 (n*=*734)	N/A (n*=*0)	14 (n*=*734)
9	Head and neck cancers	2 (n*=*24)	1 (n*=*464)	3 (n*=*488)
10	Lung cancer	10 (n*=*593)	2 (n*=*561)	12 (n*=*1154)
11	Sarcoma	3 (n*=*184)	N/A (n*=*0)	3 (n*=*184)
12	Skin cancer	1 (n*=*37)	3 (n*=*450)	4 (n*=*487)
13	Sequelae or other cancers	13 (n*=*572)	2 (n*=*507)	15 (n*=*1079)
**Total**	**13 oncology areas**	**75 (n*=*3713)**	**26 (n*=*5773)**	**101 (n*=*9486)**

The table shows the allocation of the 101 ESMO, NICE and NICE CPGs for each cancer site and with the total number of references provided in brackets.

CNS, central nervous system; CPGs, clinical practice guidelines; ESMO, European Society for Medical Oncology; N/A, none available; NICE, National Institute for Health and Care Excellence; SIGN, Scottish Intercollegiate Guidelines Network.

Each CPG citation was then downloaded as a full record from the Web of Science (this is a tab delimited text file format). These text files were then processed using Visual Basic Application programs (Evaluametrics, St Albans, England). For an example, see online supplementary appendix B. The CPGs from NICE and SIGN were grouped and compared with those from ESMO.

Unless stated below, independent sample, unpaired Student’s t-tests have been used to compare groups of citations or CPGs. The SPSS Statistics V.24.0 software was used for statistical hypotheses testing regarding: (1) country contribution in the evidence base; (2) publication year of cited research papers; (3) chronological research to reporting gap; (4) research level (RL) of papers; (5) cancer site applications; and (6) cancer research domains.

Addresses of contributing authors were available for 9391 out of the 9486 research papers (99%). Multinational citations were calculated on a fractional basis. The publication year of the citation was used to establish the interval between cited research and date of CPG. Pivot tables were used to count published papers by year and organisational body.

For journal titles, the RL of the cited papers was calculated based on keywords in the title, and of the journals in which they were published, using the system of Lewison and Paraje.^23^ Each cited paper is given a single value on a scale from 1.0=clinical to 4.0=basic science. The same process was repeated to get the average RL value for the journals in which these papers were cited. The mean RL for CPG citations was calculated by the following formula^137^:

$$\begin{array}{ll} RL= & \left(N(clinical)+4N(basic)–2.5*N(both))/(N(clinical\right)\\ & +N(basic)-N(both)) \end{array}$$

The cancer sites application macro was used to allocate one or more of the 13 tumour sites to each citation. Some papers did not specify a site and were grouped under ‘Sequelae or o*ther cancers’* together with very low frequency cancer sites.

The burden of disease was measured in disability-adjusted life-years (DALYs) for 2012 provided by the WHO.^24^ For each cancer site, this was taken as the percentage of total malignant neoplasm DALYs. Data on DALYs for sequelae, cancers of unknown origin, malignancies of the CNS, endocrine and neuroendocrine cancers, and sarcomas were grouped under ‘Sequalae or other cancers’, making the total of nine comparable groups by cancer site and disease burden.

The CPG citations were compared with the European oncology research papers, between 2002–2013. These were identified from the Web of Science using the Science Citation Index Expanded with a complex filter that contained 11 search statements; four listing 185 specialist journals in combination with seven listing 323 title words or phrases with no date restrictions.

## Results

There were 9486 citations in 101 CPGs. Distribution by cancer sites and CPG development body is summarised in table 1.

### Countries’ contributions in the evidence base

**Figure 2:**
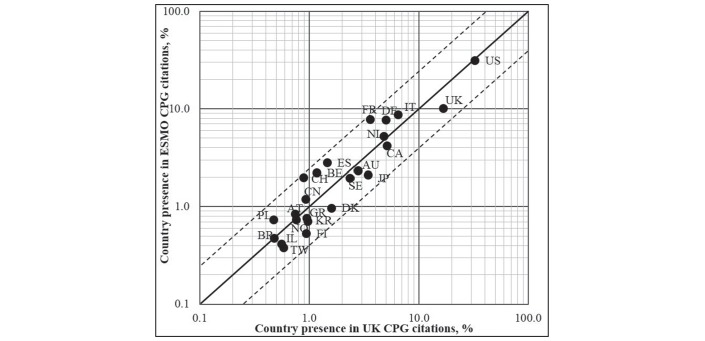
For 24 countries with more than 20 citations, this figure compares contributions to ESMO and the UK cancer CPGs. Solid line of identity indicates equal proportionate contributions. The dotted lines represent a 2.5-fold deviation from identity: χ^2^=1.0, n*=*23 and P*<*0.05. Country ISO codes: AT, Austria; AU, Australia; BE, Belgium; BR, Brazil; CA, Canada; CH, Switzerland; CN, China; DE, Germany; DK, Denmark; ES, Spain; FI, Finland; FR, France; GR, Greece; IL, Israel; IT, Italy; JP, Japan; KR, South Korea; NL, The Netherlands; NO, Norway; PL, Poland; SE, Sweden; TW, Taiwan; UK, United Kingdom; US, United States. Countries in the upper left quadrant are skewed towards ESMO, while countries on the lower right quadrant are favoured by UK CPGs. CPG, clinical practice guideline; ESMO, European Society for Medical Oncology.

The USA was the biggest contributor to the citations in the evidence base across all oncology CPGs (see figure 2). Country ranks for the ESMO CPGs were then China and mostly Western Europe (Austria, Italy, Germany, France, Spain, Belgium, Switzerland and Poland). For the UK CPGs, the greatest country contribution following the USA, were the UK, Canada, Australia, Japan, Israel, South Korea, Taiwan, Italy, the Scandinavian countries (Denmark, Sweden and Finland) and Greece.

Inspection of the residual differences suggests that for NICE and SIGN, there is a positive skew towards literature from the UK and away from France, Spain, Belgium and Switzerland, as the most extreme examples of disproportionality.

### Publication year of cited papers

**Figure 3:**
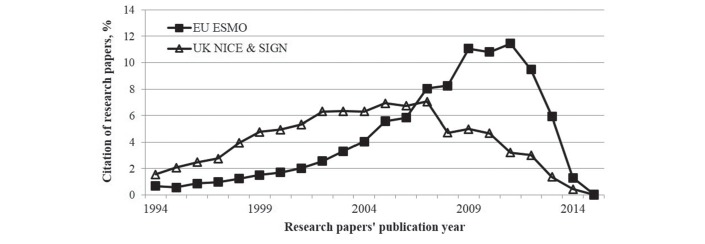
The percentage (%) distribution of cited research papers by the year of their publication (solid boxes published between 2009 and 2015 for ESMO CPGs; open triangles, n*=*13 from NICE published in 2009–2015 and n*=*13 from SIGN published in 2005–2014). The cumulated difference between the 22 annual data points is statistically significant (P*<*0.05). CPG, clinical practice guideline; ESMO, European Society for Medical Oncology; NICE, National Institute for Health and Care Excellence; SIGN, Scottish Intercollegiate Guidelines Network.

The UK set of CPGs have a flatter distribution of citations than ESMO during the 22 year feasible period of observation (see figure 3).

### Research to reporting gap

**Figure 4:**
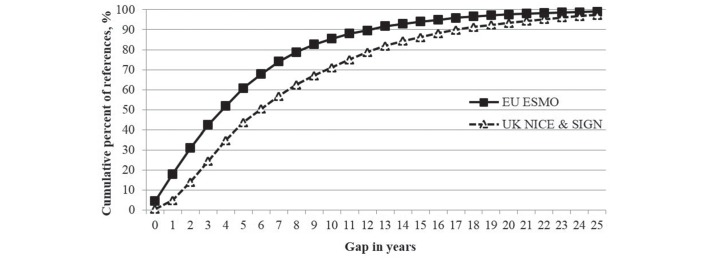
The publication gap in years for the cumulative percent of cited research papers in the ESMO and the UK overall cancer clinical guidelines, with the difference between the two sets of data points being statistically significant (P*<*0.05). ESMO, European Society for Medical Oncology; NICE, National Institute for Health and Care Excellence; SIGN, Scottish Intercollegiate Guidelines Network.

The greatest volume of cited references on ESMO CPGs (13.5%) have a very short reporting gap of only 1 year from when research was conducted to when cited in the guidelines, while those on the UK CPGs (10.5%) have a bigger gap of 4 years (results not shown). The cumulative percentages of the cited references with the gap in years are shown in figure 4. The mean gap of published research to reporting in a clinical guideline is 5.8 years for ESMO and 8.4 years for UK CPGs.

### RL of papers and journals of cited papers

The mean RL of the journals was very similar between the ESMO and UK NICE and SIGN, and had a trend towards the basic, not the clinical, end of the scale. The difference in the results was not statistically significant (P*>*0.05) in either CPG set.

In contrast to the journals, the majority of the oncology citations had an RL that was more clinical rather than basic, with a ratio of 10:1 for ESMO and 16:1 for UK CPGs (table 2). The differences in the average RL papers’ values between ESMO and NICE and SIGN were statistically significant between the CPG groups. The UK CPGs cited slightly more clinical research papers compared with the ESMO ones.

**Table 2:** The research level of the cited papers and the cited journals they are published in (85% of all citations had enough data for analysis)

References	Clinical	Basic	Both	Total assessed	RL paper	Median RL journal	Mean RL journal
ESMO	3043	321	214	3150	1.20*	1.31	1.55
UK NICE and SIGN	5259	324	244	4965	1.11*	1.27	1.41

*P*<*0.05 between CPG groups.

CPG, clinical practice guideline; ESMO, European Society for Medical Oncology; NICE, National Institute for Health and Care Excellence; RL, research level; SIGN, Scottish Intercollegiate Guidelines Network.

### Cancer sites application

**Figure 5:**
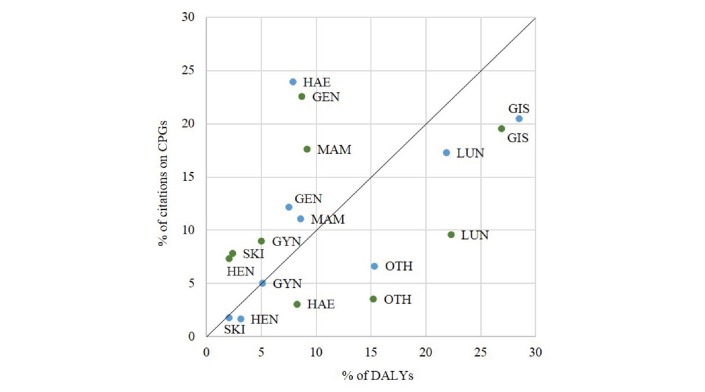
The contribution of research papers by cancer application site on the two sets of cancer clinical practice guidelines, in comparison with the corresponding disease burden (EU and UK). The nine cancer application sites were gastrointestinal carcinomas (GIS), lung cancer (LUN), sequelae or other cancer types (OTH), breast cancer (MAM), haematological malignancies (HAE), genitourinary cancers (GEN), gynaecological cancers (GYN), head and neck cancers (HEN), sarcomas and skin cancer (SKI). On GIS: EU DALYs to ESMO papers’ difference and UK DALYs to NICE and SIGN papers: P*>*0.05; on MAM: EU DALYs to ESMO papers’ difference P*>*0.05 but UK DALYs and NICE and SIGN papers: P*<*0.05; on HAE: EU DALYs and ESMO, and UK DALYs to NICE and SIGN papers: P*<*0.01 in each case. For this purpose, two-tailed paired sample t-tests were used to compare citations on CPGs with the disease burden in the corresponding region (EU and UK). Blue dots: ESMO, Green dots: NICE & SIGN. DALYs, disability-adjusted life-years; ESMO, European Society for Medical Oncology; NICE, National Institute for Health and Care Excellence; SIGN, Scottish Intercollegiate Guidelines Network.

The citations were sorted based on the cancer site according to the anatomical or organ-specific classification (see Methods and figure 5). In total, there were nine malignant neoplasm sites (including ‘Sequelaer or other Cancers’) that could be compared with the disease burden measured in DALYs.

Overall, there was a better correlation between EU malignant neoplasm DALYs and cancer research output for ESMO than the UK (except for haematological cancers). There are three observed patterns when comparing research citations with disease burden (DALYs). The item data are found in online supplementary appendix C, table S1 for EU and table S2 for UK values.

The first pattern is seen for gastrointestinal carcinomas (GIS), lung cancer (LUN) and other cancer types (OTH). For these, it appears that the proportions of disease burden, as measured by WHO DALYs, are greater than the research papers cited on this CPG topic. This is true for both ESMO and the UK NICE and SIGN CPGs. For example, there is greater burden from GIS cancers in comparison with the research attention that is being cited, which include stomach, liver and colon cancer for the EU and UK regions, out of all carcinomas. This disproportionality was the same for both CPG groups.

The second pattern is characterised by breast (MAM), gynaecological (GYN), head and neck (HEN), genitourinary (GEN) and skin (including melanoma) (SKI) cancers. For these cancer sites, the proportion of cited research exceeds the proportion of DALYs-defined disease burden for the UK. This is not true for ESMO. On MAM, research citations are contributing about 11% to ESMO CPGs, while about 9% of the European population is suffering from breast cancer, but this difference is not statistically significant (P*>*0.05). In contrast, for the UK, breast cancer research citations on CPGs are twice as much (18%) as the actual burden of disease in the UK (9%), with results being statistically significant (P*<*0.05).

A third pattern is observed only for haematological cancers, where there is a great research emphasis from ESMO clinical guidelines (24%), eightfold larger than the UK (~3%), despite a similar disease burden (7%) (P*<*0.01).

### Cancer research domains

**Figure 6:**
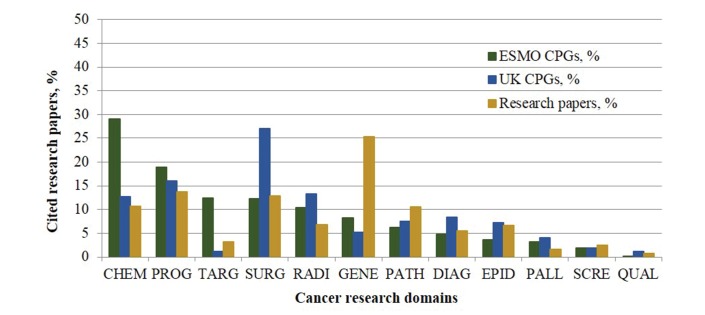
The percentage (%) contribution of the cited research papers on all the oncology cancer research domains from the citations in ESMO, the UK cancer clinical guidelines and the European research outputs (n*=*282,055) in 2002–2013. Two-tailed simple independent samples t-tests were performed to test for statistical significance between the research domains’ focus on the ESMO and UK separately. Also, two-tailed dependent samples t-tests were carried out for each group (ESMO and UK NICE and SIGN) to compare the percentage of cited research on CPGs with country research output assessment, only for the five main outstanding results. For CHEM: P*<*0.05; GENE: P*<*0.05; and SURG: P*<*0.01. The research domain codes are: CHEM, chemotherapy, DIAG, diagnosis, EPID, epidemiology, GENE, genetics, PATH, pathology, PALL, palliative care, PROG, prognosis, QUAL, quality of life research, RADI, radiotherapy, SCRE, screening, SURG, surgery and TARG, targeted therapy. CPGs, clinical practice guidelines; ESMO, European Society for Medical Oncology; NICE, National Institute for Health and Care Excellence; SIGN, Scottish Intercollegiate Guidelines Network.

Figure 6 shows the cited research for each of the 12 cancer research domains for the three sets of research papers: citations on EU ESMO CPGs, UK NICE and SIGN CPGs and European oncology research papers. The five main research domains were chemotherapy, targeted therapy, surgery, genetics and radiation therapy.

Chemotherapy was over-represented by a threefold in ESMO CPGs (29%) compared with the actual European output in oncology (11%) (P*<*0.01). The UK citations on CPGs are more proportionate (12%) as shown in figure 6.

For targeted therapies, a similar disproportion of the ESMO citations (12.3%) is observed, compared with 1.1% in the UK CPGs and 3.2% in the European output (P*<*0.01). The UK CPGs cite 2.5 times more research on surgery (27%) than ESMO (12.2%) or the EU output (12.8%) (P*<*0.01).

For radiation therapy, a statistically significant disproportion (P*<*0.05) is seen between the UK CPGs (14%), the European output (7%) and ESMO (10%). Finally, genetics research comprises a much higher proportion of the European oncology literature (19%) than the research citations in either ESMO (8%) or UK CPGs (6%) (P*<*0.01).

## Discussion

Using proprietary macros, ESMO and the UK CPGs in oncology were compared on their underlying evidence bases. All oncology guidelines were influenced by research from the USA. The ESMO CPGs also appear to be influenced mainly by research from Western Europe. The UK CPGs development is influenced by research conducted in the UK itself, Scandinavian countries, Canada, Australia, Japan, Israel, South Korea and Taiwan.

During a 22-year period, the UK CPGs on cancer indicate a preference for older and more established clinical research in comparison with European guidelines that cite newer and experimental research. Out of the 12 cancer research domains, it is evident that ESMO CPGs favour research on chemotherapy and targeted treatments, while the UK NICE and SIGN ones emphasise surgery. The UK CPGs are slightly more clinical than the ones from ESMO, and focus on screening, radiotherapy or surgery, with the results being statistically significant.

There is a preference from ESMO on more recent research, which is especially evident from 2008 and a peak in 2012, prior to declining. In contrast, the UK NICE and SIGN development bodies present a delay in the uptake of more recent evidence in the guidelines with the research papers having a wider gap from publication to citation. This mismatch of research interests corresponds to that between academia on theoretical frameworks, and clinical practice.^25^ This temporal effect may also be because the ESMO cancer guidelines are more recent than the UK ones and cite newer research and hence, it is more likely that these research publications have greater international collaboration or even integrate more evidence-based recommendations.

Strategies of guidelines’ maintenance have been debated.^12 26 27^ To find common ground between producing a timely and scientifically valid and rigorous clinical guideline, a pragmatic methodological compromise is needed.^28 29^ A suggestion that the CPGs should be reassessed for validity every 3 years^12^ has been put forward and also challenged due to the use of a heterogeneous set of guidelines with variations in quality.^30^ This reporting preference can be perhaps justified that European cancer experts developing the ESMO CPGs cite research from Western Europe, as there are many Pan-European collaborations between the various institutions. An international collaboration is needed to exchange methodological information and avoid duplicated efforts.^31 32^

One limitation of this work is that the assessment of references for classification used for the CPGs was based on a standard anatomical categorisation of the various cancers as provided by the clinical guideline bodies. However, such grouping could hide recommendations based on genetic or gender differences in the population. This is pertinent to the use of CPGs that provide no substitute to the complex clinical decision-making processes.^19 33 34^ The systematic collection, analysis and presentation of references forming the evidence base of the cancer clinical guidelines from ESMO, NICE and SIGN highlighted in this work, can provide the prototype for further research on the assessment of individual cancer sites such as breast cancer or haematology.

This study demonstrates that CPG heterogeneity begins with heterogeneity of the evidence bases. We agree with a previous study^19^ that suggested that diverse guidelines might be needed to meet divergent demands. A previous study demonstrated that the percentage of the cited papers in 15 CPGs (different topics including cancer) from UK bodies were 36% from US papers and 25% from the UK, followed by Canada 7% and Japan 2%.^11^ This form of selective reporting bias is evident with other research that shows that the work of the UK, Denmark, Ireland and Sweden were overcited in UK oncology clinical guidelines.^15^ This is the first time that a similar influence is shown for the ESMO clinical guidelines.

There is a potential solution for bias that threatens validity in clinical research, when fixed rules restrict comprehensive systematic review.^35^ It is known that papers cited on clinical guidelines are very clinical rather than basic.^10 11^ A systematic methodology for clinical guideline development is regarded as demanding, causing guideline developers to revert to consensus-based or expert-based models instead.^26 34 36 37^

Divergent demands may well justify this differential research impact in clinical guideline development. This study showed that ESMO, NICE and SIGN differ in their evidence base, and healthcare professionals should be aware of this heterogeneity when using CPGs as part of effective decision-making of tailored treatments to patients, irrespective of geographic location across Europe.
