# Supplementary material for: Differential research impact in cancer practice guidelines’ evidence base: lessons from ESMO, NICE and SIGN
Source: ESMO Open. 2018 Jan 6;3(1):e000258. doi: 10.1136/esmoopen-2017-000258 (PMC5757472; doi:10.1136/esmoopen-2017-000258)
Supplement: Supplementary file 1 [file esmoopen-2017-000258supp001.pdf]

Appendix

Appendix A – Bibliography

ESMO Clinical Practice Guideline References

1. Fizazi, K., Greco, F.A., Pavlidis, N., Daugaard, G., Oien, K. and Pentheroudakis, G., 2015. Cancers of unknown primary site: ESMO Clinical Practice Guidelines for diagnosis, treatment and follow-up. *Annals of Oncology*, *26*(suppl 5), pp.v133-v138. Available online: https://annonc.oxfordjournals.org//content/26/suppl_5/v133.full.pdf+html

2. Berruti, A., Baudin, E., Gelderblom, H., Haak, H.R., Porpiglia, F., Fassnacht, M., Pentheroudakis, G. and ESMO Guidelines Working Group, 2012. Adrenal cancer: ESMO Clinical Practice Guidelines for diagnosis, treatment and follow-up. *Annals of Oncology*, *23*(suppl 7), pp.vii131-vii138. Available online: https://annonc.oxfordjournals.org/content/23/suppl_7/vii131.full.pdf+html

3. Pacini, F., Castagna, M.G., Brilli, L., Pentheroudakis, G. and ESMO Guidelines Working Group, 2012. Thyroid cancer: ESMO Clinical Practice Guidelines for diagnosis, treatment and follow-up. *Annals of Oncology*,*23*(suppl 7), pp.vii110-vii119. Available online: https://annonc.oxfordjournals.org/content/23/suppl_7/vii110.full.pdf+html

4. Ducreux, M., Cuhna, A.S., Caramella, C., Hollebecque, A., Burtin, P., Goéré, D., Seufferlein, T., Haustermans, K., Van Laethem, J.L., Conroy, T. and Arnold, D., 2015. Cancer of the pancreas: ESMO Clinical Practice Guidelines for diagnosis, treatment and follow-up. *Annals of Oncology*, *26*(suppl 5), pp.v56-v68. Available online: https://annonc.oxfordjournals.org/content/26/suppl_5/v56.full.pdf+html

5. Van Cutsem, E., Cervantes, A., Nordlinger, B. and Arnold, D., 2014. Metastatic colorectal cancer: ESMO Clinical Practice Guidelines for diagnosis, treatment and follow-up. *Annals of oncology*, p.mdu260. Available online: https://annonc.oxfordjournals.org/content/25/suppl_3/iii1.full.pdf+html

6. Glynne-Jones, R., Nilsson, P.J., Aschele, C., Goh, V., Peiffert, D., Cervantes, A. and Arnold, D., 2014. Anal cancer: ESMO–ESSO–ESTRO clinical practice guidelines for diagnosis, treatment and follow-up. *Radiotherapy and Oncology*, *111*(3), pp.330-339. Available online: https://annonc.oxfordjournals.org/content/25/suppl_3/iii10.full.pdf+html

7. Stahl, M., Mariette, C., Haustermans, K., Cervantes, A., Arnold, D. and ESMO Guidelines Working Group, 2013. Oesophageal cancer: ESMO Clinical Practice Guidelines for diagnosis, treatment and follow-up. *Annals of oncology*, *24*(suppl 6), pp.vi51-vi56. Available online: https://annonc.oxfordjournals.org/content/24/suppl_6/vi51.full.pdf+html

8. Waddell, T., Verheij, M., Allum, W., Cunningham, D., Cervantes, A. and Arnold, D., 2013. Gastric cancer: ESMO–ESSO–ESTRO Clinical Practice Guidelines for diagnosis, treatment and follow-up. *Annals of Oncology*,*24*(suppl 6), pp.vi57-vi63. Available online: https://annonc.oxfordjournals.org/content/24/suppl_6/vi57.full.pdf+html

9. Labianca, R., Nordlinger, B., Beretta, G.D., Mosconi, S., Mandala, M., Cervantes, A., Arnold, D. and ESMO Guidelines Working Group, 2013. Early colon cancer: ESMO Clinical Practice Guidelines for diagnosis, treatment and follow-up. *Annals of oncology*, *24*(suppl 6), pp.vi64-

vi72. Available online:
https://annonc.oxfordjournals.org/content/24/suppl_6/vi64.full.pdf+html

10. Balmaña, J., Balaguer, F., Cervantes, A., Arnold, D. and ESMO Guidelines Working Group, 2013. Familial risk-colorectal cancer: ESMO clinical practice guidelines. *Annals of oncology*, p.mdt209. Available online: https://annonc.oxfordjournals.org/content/24/suppl_6/vi73.full.pdf+html

11. Glimelius, B., Tiret, E., Cervantes, A., Arnold, D. and ESMO Guidelines Working Group, 2013. Rectal cancer: ESMO Clinical Practice Guidelines for diagnosis, treatment and follow-up. *Annals of oncology*, *24*(suppl 6), pp.vi81-vi88. Available online: https://annonc.oxfordjournals.org/content/24/suppl_6/vi81.full.pdf+html

12. Schmoll, H.J., Van Cutsem, E., Stein, A., Valentini, V., Glimelius, B., Haustermans, K., Nordlinger, B., Van de Velde, C.J., Balmana, J., Regula, J. and Nagtegaal, I.D., 2012. ESMO Consensus Guidelines for management of patients with colon and rectal cancer. a personalized approach to clinical decision making. *Annals of Oncology*, *23*(10), pp.2479-2516. Available online: https://annonc.oxfordjournals.org/content/23/10/2479.full.pdf+html

13. Verslype, C., Rosmorduc, O., Rougier, P. and ESMO Guidelines Working Group, 2012. Hepatocellular carcinoma: ESMO–ESDO Clinical Practice Guidelines for diagnosis, treatment and follow-up. *Annals of oncology*,*23*(suppl 7), pp.vii41-vii48. Available online: https://annonc.oxfordjournals.org/content/23/suppl_7/vii41.full.pdf+html

14. Eckel, F., Brunner, T., Jelic, S. and ESMO Guidelines Working Group, 2011. Biliary cancer: ESMO Clinical Practice Guidelines for diagnosis, treatment and follow-up. *Annals of oncology*, *22*(suppl 6), pp.vi40-vi44. Available online: https://annonc.oxfordjournals.org/content/22/suppl_6/vi40.full.pdf+html

15. Stupp, R., Brada, M., van den Bent, M.J., Tonn, J.C., Pentheroudakis, G. and ESMO Guidelines Working Group, 2014. High-grade glioma: ESMO Clinical Practice Guidelines for diagnosis, treatment and follow-up. *Annals of Oncology*, p.mdu050. Available online: https://annonc.oxfordjournals.org/content/25/suppl_3/iii93.full.pdf+html

16. Balmana, J., Diez, O., Rubio, I.T., Cardoso, F. and ESMO Guidelines Working Group, 2011. BRCA in breast cancer: ESMO Clinical Practice Guidelines. *Annals of oncology*, *22*(suppl 6), pp.vi31-vi34. Available online: http://annonc.oxfordjournals.org/content/21/suppl_5/v20.short

17. Cardoso, F., Harbeck, N., Fallowfield, L., Kyriakides, S., Senkus, E. and ESMO Guidelines Working Group, 2012. Locally recurrent or metastatic breast cancer: ESMO Clinical Practice Guidelines for diagnosis, treatment and follow-up. *Annals of oncology*, *23*(suppl 7), pp.vii11-vii19. Available online: http://annonc.oxfordjournals.org/content/early/2013/08/22/annonc.mdt284.full.pdf+html

18. Senkus, E., Kyriakides, S., Penault-Llorca, F., Poortmans, P., Thompson, A., Zackrisson, S. and Cardoso, F., 2013. Primary breast cancer: ESMO Clinical Practice Guidelines for diagnosis, treatment and follow-up. *Annals of Oncology*, p.mdt284. Available online: http://annonc.oxfordjournals.org/content/early/2013/08/22/annonc.mdt284.full.pdf+html

19. Cardoso, F., Costa, A., Norton, L., Senkus, E., Aapro, M., Andre, F., Barrios, C.H., Bergh, J., Biganzoli, L., Blackwell, K.L. and Cardoso, M.J., 2014. ESO-ESMO 2nd international consensus

guidelines for advanced breast cancer (ABC2). *The Breast*, *23*(5), pp.489-502. Available online: http://www.sciencedirect.com/science/article/pii/S0960977614001581

20. Parker, C., Gillessen, S., Heidenreich, A. and Horwich, A., 2015. Cancer of the prostate: ESMO Clinical Practice Guidelines for diagnosis, treatment and follow-up. *Annals of Oncology*, p.mdv222. Available online: https://annonc.oxfordjournals.org/content/26/suppl_5/v69.full.pdf+html
21. Bellmunt, J., Orsola, A., Leow, J.J., Wiegel, T., De Santis, M. and Horwich, A., 2014. Bladder cancer: ESMO Practice Guidelines for diagnosis, treatment and follow-up. *Annals of Oncology*, *25*(suppl 3), pp.iii40-iii48. Available online: https://annonc.oxfordjournals.org/content/25/suppl_3/iii40.full.pdf+html
22. Escudier, B., Porta, C., Schmidinger, M., Algaba, F., Patard, J.J., Khoo, V., Eisen, T. and Horwich, A., 2014. Renal cell carcinoma: ESMO Clinical Practice Guidelines for diagnosis, treatment and follow-up†. *Annals of Oncology*, *25*(suppl 3), pp.iii49-iii56. Available online: https://annonc.oxfordjournals.org/content/25/suppl_3/iii49.full.pdf+html
23. Van Poppel, H., Watkin, N.A., Osanto, S., Moonen, L., Horwich, A., Kataja, V. and ESMO Guidelines Working Group, 2013. Penile cancer: ESMO Clinical Practice Guidelines for diagnosis, treatment and follow-up. *Annals of oncology*, *24*(suppl 6), pp.vi115-vi124. Available online: https://annonc.oxfordjournals.org/content/24/suppl_6/vi115.full.pdf+html
24. Oldenburg, J., Fosså, S.D., Nuver, J., Heidenreich, A., Schmoll, H.J., Bokemeyer, C., Horwich, A., Beyer, J., Kataja, V. and ESMO Guidelines Working Group, 2013. Testicular seminoma and non-seminoma: ESMO Clinical Practice Guidelines for diagnosis, treatment and follow-up. *Annals of oncology*, *24*(suppl 6), pp.vi125-vi132. Available online: https://annonc.oxfordjournals.org/content/24/suppl_6/vi125.full.pdf+html
25. Horwich, A., Hugosson, J., de Reijke, T., Wiegel, T., Fizazi, K. and Kataja, V., 2013. Prostate cancer: ESMO consensus conference guidelines 2012. *Annals of oncology*, p.mds624. Available online: https://annonc.oxfordjournals.org/content/24/5/1141.full.pdf+html
26. Colombo, N., Preti, E., Landoni, F., Carinelli, S., Colombo, A., Marini, C., Sessa, C. and ESMO Guidelines Working Group, 2013. Endometrial cancer: ESMO Clinical Practice Guidelines for diagnosis, treatment and follow-up. *Annals of oncology*, *24*(suppl 6), pp.vi33-vi38. Available online: https://annonc.oxfordjournals.org/content/24/suppl_6/vi33.full.pdf+html
27. Seckl, M.J., Sebire, N.J., Fisher, R.A., Golfier, F., Massuger, L., Sessa, C. and ESMO Guidelines Working Group, 2013. Gestational trophoblastic disease: ESMO Clinical Practice Guidelines for diagnosis, treatment and follow-up. *Annals of oncology*, *24*(suppl 6), pp.vi39-vi50. Available online: https://annonc.oxfordjournals.org/content/24/suppl_6/vi39.full.pdf+html
28. Ledermann, J.A., Raja, F.A., Fotopoulou, C., Gonzalez-Martin, A., Colombo, N., Sessa, C. and ESMO Guidelines Working Group, 2013. Newly diagnosed and relapsed epithelial ovarian carcinoma: ESMO Clinical Practice Guidelines for diagnosis, treatment and follow-up. *Annals of oncology*,*24*(suppl 6), pp.vi24-vi32. Available online: https://annonc.oxfordjournals.org/content/24/suppl_6/vi24.full.pdf+html
29. Colombo, N., Peiretti, M., Garbi, A., Carinelli, S., Marini, C., Sessa, C. and ESMO Guidelines Working Group, 2012. Non-epithelial ovarian cancer: ESMO Clinical Practice Guidelines for

diagnosis, treatment and follow-up.*Annals of oncology*, *23*(suppl 7), pp.vii20-vii26. Available online: https://annonc.oxfordjournals.org/content/23/suppl_7/vii20.full.pdf+html

30. Colombo, N., Carinelli, S., Colombo, A., Marini, C., Rollo, D., Sessa, C. and ESMO Guidelines Working Group, 2012. Cervical cancer: ESMO Clinical Practice Guidelines for diagnosis, treatment and follow-up. *Annals of oncology*, *23*(suppl 7), pp.vii27-vii32. Available online: https://annonc.oxfordjournals.org/content/23/suppl_7/vii27.full.pdf+html

31. Chan, A.T.C., Grégoire, V., Lefebvre, J.L., Licitra, L., Hui, E.P., Leung, S.F., Felip, E. and EHNS–ESMO–ESTRO Guidelines Working Group, 2012. Nasopharyngeal cancer: EHNS–ESMO–ESTRO Clinical Practice Guidelines for diagnosis, treatment and follow-up. *Annals of oncology*, *23*(suppl 7), pp.vii83-vii85. Available online: https://annonc.oxfordjournals.org/content/23/suppl_7/vii83.full.pdf+html

32. Grégoire, V., Lefebvre, J.L., Licitra, L., Felip, E. and EHNS–ESMO–ESTRO Guidelines Working Group, 2010. Squamous cell carcinoma of the head and neck: EHNS–ESMO–ESTRO Clinical Practice Guidelines for diagnosis, treatment and follow-up. *Annals of oncology*, *21*(suppl 5), pp.v184-v186. Available online: https://annonc.oxfordjournals.org/content/21/suppl_5/v184.full.pdf+html

33. Dummer, R., Hauschild, A., Guggenheim, M., Keilholz, U., Pentheroudakis, G. and ESMO Guidelines Working Group, 2012. Cutaneous melanoma: ESMO Clinical Practice Guidelines for diagnosis, treatment and follow-up.*Annals of Oncology*, *23*(suppl 7), pp.vii86-vii91. Available online: https://annonc.oxfordjournals.org/content/26/suppl_5/v126.full.pdf+html

34. Öberg, K., Hellman, P., Ferolla, P., Papotti, M. and ESMO Guidelines Working Group, 2012. Neuroendocrine bronchial and thymic tumors: ESMO Clinical Practice Guidelines for diagnosis, treatment and follow-up. *Annals of oncology*, *23*(suppl 7), pp.vii120-vii123. Available online: https://annonc.oxfordjournals.org/content/23/suppl_7/vii120.full.pdf+html

35. Öberg, K., Knigge, U., Kwekkeboom, D., Perren, A. and ESMO Guidelines Working Group, 2012. Neuroendocrine gastro-entero-pancreatic tumors: ESMO Clinical Practice Guidelines for diagnosis, treatment and follow-up.*Annals of Oncology*, *23*(suppl 7), pp.vii124-vii130. Available online: https://annonc.oxfordjournals.org/content/23/suppl_7/vii124.full.pdf

36. ESMO/European Sarcoma Network Working Group, 2014. Soft tissue and visceral sarcomas: ESMO Clinical Practice Guidelines for diagnosis, treatment and follow-up. *Annals of Oncology*, *25*(suppl 3), pp.iii102-iii112. Available online: https://annonc.oxfordjournals.org/content/25/suppl_3/iii102.full.pdf+html

37. ESMO/European Sarcoma Network Working Group, 2014. Bone sarcomas: ESMO Clinical Practice Guidelines for diagnosis, treatment and follow-up.*Annals of Oncology*, *25*(suppl 3), pp.iii113-iii123. Available online: https://annonc.oxfordjournals.org/content/25/suppl_3/iii113.full.pdf+html

38. ESMO/European Sarcoma Network Working Group, 2014. Gastrointestinal stromal tumours: ESMO Clinical Practice Guidelines for diagnosis, treatment and follow-up. *Annals of oncology: official journal of the European Society for Medical Oncology/ESMO*, *25*, p.iii21. Available online: https://annonc.oxfordjournals.org/content/25/suppl_3//iii21.full.pdf+html

39. Stahel, R.A., Weder, W., Lievens, Y.O.L.A.N.D.E., Felip, E. and ESMO Guidelines Working Group, 2010. Malignant pleural mesothelioma: ESMO Clinical Practice Guidelines for

diagnosis, treatment and follow-up. *Annals of oncology*, *21*(suppl 5), pp.v126-v128. Available online: https://annonc.oxfordjournals.org/content/21/suppl_5/v126.full.pdf+html

40. Peters, S., Adjei, A.A., Gridelli, C., Reck, M., Kerr, K., Felip, E.E.S.M.O. and ESMO Guidelines Working Group, 2012. Metastatic non-small-cell lung cancer (NSCLC): ESMO Clinical Practice Guidelines for diagnosis, treatment and follow-up. *Annals of Oncology*, *23*(suppl 7), pp.vii56-vii64. Available online: http://annonc.oxfordjournals.org/content/23/suppl_7/vii56.full.pdf+html

41. Früh, M., De Ruysscher, D., Popat, S., Crinò, L., Peters, S., Felip, E. and ESMO Guidelines Working Group, 2013. Small-cell lung cancer (SCLC): ESMO Clinical Practice Guidelines for diagnosis, treatment and follow-up.*Annals of oncology*, p.mdt178. available online: https://annonc.oxfordjournals.org/content/early/2013/06/26/annonc.mdt178.full.pdf+html

42. Vansteenkiste, J., De Ruysscher, D., Eberhardt, W.E.E., Lim, E., Senan, S., Felip, E. and Peters, S., 2013. Early and locally advanced non-small-cell lung cancer (NSCLC): ESMO Clinical Practice Guidelines for diagnosis, treatment and follow-up. *Annals of oncology*, p.mdt241. Available online: http://annonc.oxfordjournals.org/content/early/2013/07/15/annonc.mdt241.full.pdf+html

43. Vansteenkiste, J., Crinò, L., Dooms, C., Douillard, J.Y., Faivre-Finn, C., Lim, E., Rocco, G., Senan, S., Van Schil, P., Veronesi, G. and Stahel, R., 2014. 2nd ESMO Consensus Conference on Lung Cancer: early-stage non-small-cell lung cancer consensus on diagnosis, treatment and follow-up. *Annals of Oncology*, p.mdu089. Available online: https://annonc.oxfordjournals.org/content/early/2014/05/07/annonc.mdu089.full.pdf+html

44. Felip, E., Gridelli, C., Baas, P., Rosell, R. and Stahel, R., 2011. Metastatic non-small-cell lung cancer: consensus on pathology and molecular tests, first-line, second-line, and third-line therapy 1st ESMO Consensus Conference in Lung Cancer; Lugano 2010. *Annals of oncology*, p.mdr150. Available online: http://annonc.oxfordjournals.org/content/early/2011/05/20/annonc.mdr150.full.pdf+html

45. Reck, M., Popat, S., Reinmuth, N., De Ruysscher, D., Kerr, K.M. and Peters, S., 2014. Metastatic non-small-cell lung cancer (NSCLC): ESMO Clinical Practice Guidelines for diagnosis, treatment and follow-up. *Annals of oncology*, p.mdu199. Available online: http://annonc.oxfordjournals.org/content/early/2014/08/11/annonc.mdu199.full.pdf+html

46. Kerr, K.M., Bubendorf, L., Edelman, M.J., Marchetti, A., Mok, T., Novello, S., O'Byrne, K., Stahel, R., Peters, S., Felip, E. and Stahel, R., 2014. Second ESMO consensus conference on lung cancer: pathology and molecular biomarkers for non-small-cell lung cancer. *Annals of Oncology*, *25*(9), pp.1681-1690. Available online: http://annonc.oxfordjournals.org/content/25/9/1681.full.pdf+html

47. Besse, B., Adjei, A., Baas, P., Meldgaard, P., Nicolson, M., Paz-Ares, L., Reck, M., Smit, E.F., Syrigos, K., Stahel, R. and Felip, E., 2014. 2nd ESMO Consensus Conference on Lung Cancer: non-small-cell lung cancer first-line/second and further lines of treatment in advanced disease. *Annals of Oncology*, p.mdu123. Available online: https://annonc.oxfordjournals.org/content/early/2014/05/11/annonc.mdu123.full.pdf+html

48. Stahel, R., Thatcher, N., Früh, M., Le Péchoux, C., Postmus, P.E., Sorensen, J.B. and Felip, E., 2011. 1st ESMO Consensus Conference in lung cancer; Lugano 2010: small-cell lung

cancer. *Annals of Oncology*, p.mdr313. Available online: https://annonc.oxfordjournals.org/content/early/2011/07/04/annonc.mdr313.full.pdf+html

49. Crawford, J., Caserta, C., Roila, F. and ESMO Guidelines Working Group, 2010. Hematopoietic growth factors: ESMO Clinical Practice Guidelines for the applications. *Annals of oncology*, *21*(suppl 5), pp.v248-v251. Available online: http://annonc.oxfordjournals.org/content/21/suppl_5/v248.full.pdf+html

50. De Naurois, J., Novitzky-Basso, I., Gill, M.J., Marti, F.M., Cullen, M.H., Roila, F. and ESMO Guidelines Working Group, 2010. Management of febrile neutropenia: ESMO clinical practice guidelines. *Annals of Oncology*, *21*(suppl 5), pp.v252-v256. Available online: http://annonc.oxfordjournals.org/content/21/suppl_5/v252.full.pdf+html

51. Roila, F., Herrstedt, J., Aapro, M., Gralla, R.J., Einhorn, L.H., Ballatori, E., Bria, E., Clark-Snow, R.A., Espersen, B.T., Feyer, P. and Grunberg, S.M., 2010. Guideline update for MASCC and ESMO in the prevention of chemotherapy-and radiotherapy-induced nausea and vomiting: results of the Perugia consensus conference. *Annals of Oncology*, *21*(suppl 5), pp.v232-v243. Available online: http://annonc.oxfordjournals.org/content/21/suppl_5/v232.full.pdf+html

52. Schrijvers, D., De Samblanx, H. and Roila, F., 2010. Erythropoiesis-stimulating agents in the treatment of anaemia in cancer patients: ESMO Clinical Practice Guidelines for use. *Ann Oncol*, *21*(Suppl 5), pp.v244-v247.

53. Mandala, M., Falanga, A., Roila, F. and ESMO Guidelines Working Group, 2011. Management of venous thromboembolism (VTE) in cancer patients: ESMO Clinical Practice Guidelines. *Annals of Oncology*, *22*(suppl 6), pp.vi85-vi92. Available online: http://annonc.oxfordjournals.org/content/22/suppl_6/vi85.full.pdf+html

54. Peterson, D.E., Bensadoun, R.J., Roila, F. and ESMO Guidelines Working Group, 2011. Management of oral and gastrointestinal mucositis: ESMO Clinical Practice Guidelines. *Annals of oncology*, *22*(suppl 6), pp.vi78-vi84. Available online: http://annonc.oxfordjournals.org/content/22/suppl_6/vi78.full.pdf+html

55. Curigliano, G., Cardinale, D., Suter, T., Plataniotis, G., de Azambuja, E., Sandri, M.T., Criscitiello, C., Goldhirsch, A., Cipolla, C., Roila, F. and ESMO Guidelines Working Group, 2012. Cardiovascular toxicity induced by chemotherapy, targeted agents and radiotherapy: ESMO Clinical Practice Guidelines. *Annals of oncology*, *23*(suppl 7), pp.vii155-vii166. Available online: https://annonc.oxfordjournals.org/content/23/suppl_7/vii155.full.pdf+html

56. Fidalgo, J.P., Fabregat, L.G., Cervantes, A., Margulies, A., Vidall, C., Roila, F. and ESMO Guidelines Working Group, 2012. Management of chemotherapy extravasation: ESMO–EONS Clinical Practice Guidelines. *Annals of oncology*, *23*(suppl 7), pp.vii167-vii173. Available online: http://annonc.oxfordjournals.org/content/23/suppl_7/vii167.full.pdf+html

57. Ripamonti, C.I., Santini, D., Maranzano, E., Berti, M., Roila, F. and ESMO Guidelines Working Group, 2012. Management of cancer pain: ESMO clinical practice guidelines. *Annals of oncology*, *23*(suppl 7), pp.vii139-vii154. Available online: http://annonc.oxfordjournals.org/content/23/suppl_7/vii139.full.pdf+html

58. Peccatori, F.A., Azim, H.A., Orecchia, R., Hoekstra, H.J., Pavlidis, N., Kesic, V., Pentheroudakis, G. and ESMO Guidelines Working Group, 2013. Cancer, pregnancy and fertility: ESMO Clinical Practice Guidelines for diagnosis, treatment and follow-up. *Annals of oncology*, p.mdt199.

Available online:
http://annonc.oxfordjournals.org/content/early/2013/06/26/annonc.mdt199.full.pdf+html

59. Coleman, R., Body, J.J., Aapro, M., Hadji, P., Herrstedt, J. and ESMO Guidelines Working Group, 2014. Bone health in cancer patients: ESMO Clinical Practice Guidelines. *Annals of Oncology*, p.mdu103. Available online:
http://annonc.oxfordjournals.org/content/early/2014/04/29/annonc.mdu103.full.pdf+html

60. Schrijvers, D. and Cherny, N.I., 2014. ESMO Clinical Practice Guidelines on palliative care: advanced care planning. *Annals of Oncology*, *25*(suppl 3), pp.iii138-iii142. Available online:
https://annonc.oxfordjournals.org/content/25/suppl_3/iii138.full

61. Cherny, N.I., 2014. ESMO Clinical Practice Guidelines for the management of refractory symptoms at the end of life and the use of palliative sedation.*Annals of Oncology*, *25*(suppl 3), pp.iii143-iii152. Available online:
https://annonc.oxfordjournals.org/content/25/suppl_3/iii143.full.pdf+html

62. Dreyling, M., Thieblemont, C., Gallamini, A., Arcaini, L., Campo, E., Hermine, O., Kluin-Nelemans, J.C., Ladetto, M., Le Gouill, S., Iannitto, E. and Pileri, S., 2013. ESMO Consensus conferences: guidelines on malignant lymphoma. part 2: marginal zone lymphoma, mantle cell lymphoma, peripheral T-cell lymphoma. *Annals of oncology*, *24*(4), pp.857-877. Available online: http://annonc.oxfordjournals.org/content/24/4/857.full.pdf+html

63. Eichenauer, D.A., Engert, A., André, M., Federico, M., Illidge, T., Hutchings, M. and Ladetto, M., 2014. Hodgkin's lymphoma: ESMO Clinical Practice Guidelines for diagnosis, treatment and follow-up. *Annals of Oncology*,*25*(suppl 3), pp.iii70-iii75. Available online:
https://annonc.oxfordjournals.org/content/25/suppl_3/iii70.full.pdf+html

64. Eichhorst, B., Dreyling, M., Robak, T., Montserrat, E., Hallek, M. and ESMO Guidelines Working Group, 2011. Chronic lymphocytic leukemia: ESMO Clinical Practice Guidelines for diagnosis, treatment and follow-up. *Annals of Oncology*, *22*(suppl 6), pp.vi50-vi54. Available online: https://annonc.oxfordjournals.org/content/22/suppl_6/vi50.full.pdf+html

65. Baccarani, M., Pileri, S., Steegmann, J.L., Muller, M., Soverini, S., Dreyling, M. and ESMO Guidelines Working Group, 2012. Chronic myeloid leukemia: ESMO Clinical Practice Guidelines for diagnosis, treatment and follow-up.*Annals of oncology*, *23*(suppl 7), pp.vii72-vii77. Available online:
http://annonc.oxfordjournals.org/content/23/suppl_7/vii72.full.pdf+html

66. Tilly, H., Vitolo, U., Walewski, J., da Silva, M.G., Shpilberg, O., Andre, M., Pfreundschuh, M., Dreyling, M. and ESMO Guidelines Working Group, 2012. Diffuse large B-cell lymphoma (DLBCL): ESMO Clinical Practice Guidelines for diagnosis, treatment and follow-up. *Annals of oncology*, *23*(suppl 7), pp.vii78-vii82. Available online:
http://annonc.oxfordjournals.org/content/23/suppl_7/vii78.full.pdf+html

67. Buske, C., Leblond, V., Dimopoulos, M., Kimby, E., Jäger, U., Dreyling, M. and ESMO Guidelines Working Group, 2013. Waldenström's macroglobulinaemia: ESMO Clinical Practice Guidelines for diagnosis, treatment and follow-up. *Annals of oncology*, *24*(suppl 6), pp.vi155-vi159. Available online:
https://annonc.oxfordjournals.org/content/24/suppl_6/vi155.full.pdf+html

68. Fey, M.F. and Buske, C., 2013. Acute myeloblastic leukaemias in adult patients: ESMO Clinical Practice Guidelines for diagnosis, treatment and follow-up. *Annals of oncology*, p.mdt320. Available online: https://annonc.oxfordjournals.org/content/early/2013/08/22/annonc.mdt320.full.pdf+html

69. Harousseau, J.L. and Dreyling, M., 2010. Multiple myeloma: ESMO Clinical Practice Guidelines for diagnosis, treatment and follow-up. *Ann Oncol*,*21*(Suppl 5), pp.v155-v157. Available online: http://www.kosmidisoncology.com/img/4ddc8821c34bceaf3dba4a18741acca3GUIDELINES%20FOR%20MULTIPLE%20MYELOMA.full.pdf

70. Willemze, R., Hodak, E., Zinzani, P.L., Specht, L., Ladetto, M. and ESMO Guidelines Working Group, 2013. Primary cutaneous lymphomas: ESMO Clinical Practice Guidelines for diagnosis, treatment and follow-up. *Annals of oncology*, *24*(suppl 6), pp.vi149-vi154. Available online: http://annonc.oxfordjournals.org/content/24/suppl_6/vi149.full.pdf+html

71. Zucca, E., Copie-Bergman, C., Ricardi, U., Thieblemont, C., Raderer, M., Ladetto, M. and ESMO Guidelines Working Group, 2013. Gastric marginal zone lymphoma of MALT type: ESMO Clinical Practice Guidelines for diagnosis, treatment and follow-up. *Annals of oncology*, *24*(suppl 6), pp.vi144-vi148. Available online: https://annonc.oxfordjournals.org/content/24/suppl_6/vi144.full.pdf+html

72. Ghielmini, M., Vitolo, U., Kimby, E., Montoto, S., Walewski, J., Pfreundschuh, M., Federico, M., Hoskin, P., McNamara, C., Caligaris-Cappio, F. and Stilgenbauer, S., 2012. ESMO Guidelines consensus conference on malignant lymphoma 2011 part 1: diffuse large B-cell lymphoma (DLBCL), follicular lymphoma (FL) and chronic lymphocytic leukemia (CLL). *Annals of oncology*, p.mds517. Available online: https://annonc.oxfordjournals.org/content/early/2012/11/21/annonc.mds517.full.pdf+html

73. Fenaux, P., Haase, D., Sanz, G.F., Santini, V. and Buske, C., 2014. Myelodysplastic syndromes: ESMO Clinical Practice Guidelines for diagnosis, treatment and follow-up. *Annals of Oncology*, p.mdu180. Available online: http://annonc.oxfordjournals.org/content/early/2014/07/25/annonc.mdu180.full.pdf+html

74. Dreyling, M., Ghielmini, M., Marcus, R., Salles, G., Vitolo, U. and Ladetto, M., 2014. Newly diagnosed and relapsed follicular lymphoma: ESMO Clinical Practice Guidelines for diagnosis, treatment and follow-up. *Annals of Oncology*, *25*(suppl 3), pp.iii76-iii82. Available online: https://annonc.oxfordjournals.org/content/25/suppl_3/iii76.full.pdf+html

75. Dreyling, M., Geisler, C., Hermine, O., Kluin-Nelemans, H.C., Le Gouill, S., Rule, S., Shpilberg, O., Walewski, J. and Ladetto, M., 2014. Newly diagnosed and relapsed mantle cell lymphoma: ESMO Clinical Practice Guidelines for diagnosis, treatment and follow-up. *Annals of Oncology*, *25*(suppl 3), pp.iii83-iii92. Available online: https://annonc.oxfordjournals.org/content/25/suppl_3/iii83.full.pdf+html

NICE Clinical Practice Guideline References

1. National Collaborating Centre for Cancer, 2010. Metastatic malignant disease of unknown primary origin—diagnosis and management of metastatic malignant disease of unknown primary origin. *NICE Clinical Guideline*, *104*, pp.1-38. Available online: https://www.nice.org.uk/guidance/cg104/evidence/full-guideline-134697133

2. Centre for Clinical Practice, 2011. Colonoscopic surveillance for preventing colorectal cancer in adults with ulcerative colitis, Crohn's disease or adenoma. *NICE Clinical Guideline*, *118*, pp.1-117. Available online: https://www.nice.org.uk/guidance/cg118/evidence/full-guideline-181410157
3. Centre for Clinical Practice, 2010. Barrett's oesophagus Ablative therapy for the treatment of Barrett's oesophagus. *NICE Clinical Guideline*, *106*, pp.1-96. Available online: https://www.nice.org.uk/guidance/cg106/evidence/ablative-therapy-full-guideline-134747677
4. National Collaborating Centre for Cancer, 2011. Lung cancer: diagnosis and management, Crohn's disease or adenoma. *NICE Clinical Guideline*, *121*, pp.1-198. Available online: https://www.nice.org.uk/guidance/cg121/evidence/full-guideline-181636957
5. National Collaborating Centre for Cancer, 2011. Ovarian cancer: recognition and initial management. *NICE Clinical Guideline*, *122*, pp.1-148. Available online: https://www.nice.org.uk/guidance/cg122/evidence/full-guideline-181688797
6. National Collaborating Centre for Cancer, 2011. Colorectal cancer: diagnosis and management. *NICE Clinical Guideline*, *131*, pp.1-186. Available online: https://www.nice.org.uk/guidance/cg131/evidence/full-guideline-183509677
7. National Collaborating Centre for Cancer, 2013. Familial breast cancer: classification, care and managing breast cancer and related risks in people with a family history of breast cancer. *NICE Clinical Guideline*, *164*, pp.1-265. Available online:https://www.nice.org.uk/guidance/cg164/evidence/full-guideline-190130941
8. National Collaborating Centre for Cancer, 2014. Prostate cancer: diagnosis and management. *NICE Clinical Guideline*, *175*, pp.1-478. Available online: Available online:https://www.nice.org.uk/guidance/cg175/evidence/full-guideline-191710765
9. National Collaborating Centre for Cancer, 2009. Early and locally advanced breast cancer: diagnosis and treatment. *NICE Clinical Guideline*, *80*, pp.1-193. Available online: https://www.nice.org.uk/guidance/cg80/evidence/full-guideline-242201629
10. National Collaborating Centre for Cancer, 2009. Advanced breast cancer: diagnosis and treatment. *NICE Clinical Guideline*, *81*, pp.1-122. Available online: https://www.nice.org.uk/guidance/cg81/evidence/full-guideline-242246989
11. National Collaborating Centre for Cancer, 2015. Suspected cancer: recognition and referral. *NICE Clinical Guideline*, *NG12,* pp.1-378. Available online: https://www.nice.org.uk/guidance/ng12/evidence/full-guidance-74333341
12. National Collaborating Centre for Cancer, 2015. Melanoma: assessment and management. *NICE Clinical Guideline*, *NG14,* pp.1-246. Available online: https://www.nice.org.uk/guidance/ng14/evidence/full-guideline-250314589
13. National Collaborating Centre for Cancer, 2015. Bladder cancer: diagnosis and management. *NICE Clinical Guideline*, *NG2,* pp.1-500. Available online: https://www.nice.org.uk/guidance/ng2/evidence/full-guideline-3744112

SIGN Clinical Practice Guideline References

1. Scottish Intercollegiate Guidelines Network, 2003. *Cutaneous Melanoma: A National Clinical Guideline*. Scottish Intercollegiate Guidelines Network, SIGN 72, pp: 1-55.

2. Scottish Intercollegiate Guidelines Network, 2005. *Management of transitional cell carcinoma: A National Clinical Guideline*. Scottish Intercollegiate Guidelines Network, SIGN 85, pp: 1-48.

3. Scottish Intercollegiate Guidelines Network, 2006. *Management of oesophageal and gastric cancer: A National Clinical Guideline*. Scottish Intercollegiate Guidelines Network, SIGN 87, pp: 1-74.

4. Scottish Intercollegiate Guidelines Network, 2006. *Diagnosis and management of head and neck cancer: A National Clinical Guideline*. Scottish Intercollegiate Guidelines Network, SIGN 90, pp: 1-96.

5. Scottish Intercollegiate Guidelines Network, 2008. *Management of cervical cancer: A National Clinical Guideline*. Scottish Intercollegiate Guidelines Network, SIGN 99, pp: 1-77.

6. Scottish Intercollegiate Guidelines Network, 2008. *Control of pain in adults with cancer: A National Clinical Guideline*. Scottish Intercollegiate Guidelines Network, SIGN 106, pp: 1-78.

7. Scottish Intercollegiate Guidelines Network, 2011. *Management of adult testicular germ cell tumours: A National Clinical Guideline*. Scottish Intercollegiate Guidelines Network, SIGN 124, pp: 1-70.

8. Scottish Intercollegiate Guidelines Network, 2011. *Diagnosis and management of colorectal cancer: A National Clinical Guideline*. Scottish Intercollegiate Guidelines Network, SIGN 126, pp: 1-63.

9. Scottish Intercollegiate Guidelines Network, 2013. *Long term follow up of survivors of childhood cancer: A National Clinical Guideline*. Scottish Intercollegiate Guidelines Network, SIGN 132, pp: 1-69.

10. Scottish Intercollegiate Guidelines Network, 2013. *Treatment of primary breast cancer: A National Clinical Guideline*. Scottish Intercollegiate Guidelines Network, SIGN 134, pp: 1-50.

11. Scottish Intercollegiate Guidelines Network, 2013. *Management of epithelial ovarian cancer: A National Clinical Guideline*. Scottish Intercollegiate Guidelines Network, SIGN 135, pp: 1-66.

12. Scottish Intercollegiate Guidelines Network, 2014. *Management of lung cancer: A National Clinical Guideline*. Scottish Intercollegiate Guidelines Network, SIGN 137, pp: 1-74.

13. Scottish Intercollegiate Guidelines Network, 2014. *Management of primary cutaneous squamous cell carcinoma: A National Clinical Guideline*. Scottish Intercollegiate Guidelines Network, SIGN 140, pp: 1-51.

Appendix B – Web of Science search statement example

An example of a reference cited in an ESMO breast cancer clinical guideline is provided here: Antoniou A, Pharoah PD, Narod S et al. Average risks of breast and ovarian cancer associated with BRCA1 or BRCA2 mutations detected in case Series unselected for family history: a combined analysis of 22 studies. Am J Hum Genet. 2003; 72: 1117–1130. The search statement developed was: AU=Antoniou and PY=2003 and TI=(breast AND mutations AND 22)

Appendix C – Data values

Table S1 – The data on the disability-adjusted-life-years in EU for each of the cancer sites in comparison to the research output for ESMO clinical guidelines and the research papers

Site	EU DALYs, %	ESMO CPGs site references	ESMO CPGs, %	ESMO paper site	ESMO papers, %
**GIS**	**28.50**	522	**14.06**	632	**20.47**
**LUN**	**21.87**	593	**15.97**	533	**17.27**
**OTH**	**15.33**	962	**25.91**	205	**6.64**
**MAM**	**8.59**	271	**7.30**	342	**11.08**
**HAE**	**7.88**	734	**19.77**	739	**23.94**
**GEN**	**7.53**	385	**10.37**	376	**12.18**
**GYN**	**5.10**	185	**4.98**	155	**5.02**
**HEN**	**3.11**	24	**0.65**	51	**1.65**
**SKI**	**2.09**	37	**1.00**	54	**1.75**
**TOTAL**	**100.00**	**3713**	**100.00**	**3087**	**100.00**

Table S2 – The data on the disability-adjusted-life-years in UK for each of the cancer sites in comparison to the research output for UK clinical guidelines and the research paper

Site	UK DALYs, %	UK CPGs site references	UK CPGs, %	UK paper site	UK papers, %
**GIS**	**26.89**	788	**13.65**	997	**19.55**
**LUN**	**22.28**	561	**9.72**	488	**9.57**
**OTH**	**15.22**	724	**12.54**	181	**3.55**
**MAM**	**9.18**	966	**16.73**	898	**17.61**
**HAE**	**8.26**	0	**0.00**	156	**3.06**
**GEN**	**8.69**	1334	**23.11**	1151	**22.57**
**GYN**	**5.03**	486	**8.42**	457	**8.96**
**HEN**	**2.09**	464	**8.04**	373	**7.32**
**SKI**	**2.36**	450	**7.79**	398	**7.81**
**TOTAL**	**100.00**	**5773**	**100.00**	**5099**	**100.00**
